# Unraveling the Applicability of LbL Coatings for Drug Delivery in Dental Implant-Related Infection Treatment

**DOI:** 10.1021/acsbiomaterials.4c01037

**Published:** 2024-11-30

**Authors:** Marta Maria Alves Pereira, Rodolfo Piazza, Amanda Paino Santana, Valentim Adelino Ricardo Barão, Samuel Santana Malheiros, Jeroen J. J. P. van den Beucken, Rafael Scaf de Molon, Erica Dorigatti de Avila

**Affiliations:** † Department of Dental Materials and Prosthodontics, São Paulo State University (UNESP), School of Dentistry, Araraquara, São Paulo 14801-903, Brazil; ‡ Department of Physical Chemistry, São Paulo State University (UNESP), Institute of Chemistry, Araraquara, São Paulo 14801-970, Brazil; § Department of Dental Materials and Prosthodontics, São Paulo State University (UNESP), School of Dentistry, Araçatuba, São Paulo 16015-050, Brazil; ∥ Department of Prosthodontics and Periodontology, Piracicaba Dental School, Universidade Estadual de Campinas (UNICAMP), Piracicaba, São Paulo 13414-903, Brazil; ⊥ Dentistry-Regenerative Biomaterials, Radboudumc ,Nijmegen, Gelderland 6500 HB, The Netherlands; # Department of Diagnostic and Surgery, São Paulo State University (UNESP), School of Dentistry, Araçatuba, São Paulo 16015-050, Brazil

**Keywords:** antimicrobial coating, layer-by-layer system, drug delivery system, dental implants, peri-implantitis

## Abstract

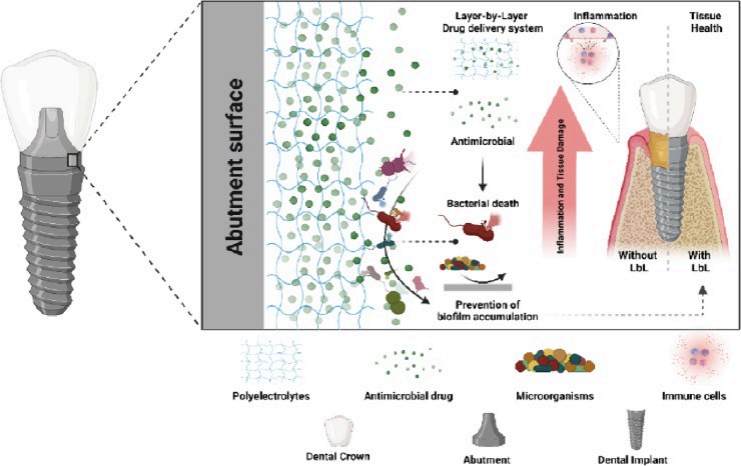

Peri-implantitis is an inflammatory condition caused by bacterial biofilms adhered on dental implant surfaces that cause progressive tissue destruction from the host’s inflammatory response. The adverse effects of peri-implantitis progression can go beyond just losing the implant. This highlights the importance of implementing strategies to stabilize disease in the short term. Layer-by-layer (LbL) assembly is a promising avenue in the field of peri-implantitis management due to its applicability with a variety of substances, in addition to being an easy, versatile, and flexible process for multilayer formation to act directly in the affected site. In this Review, our objective is to offer comprehensive chemical and biological insights into the LbL system, clarifying its specific application as antimicrobial coatings, with concern for the physical site and purpose. Additionally, we delve deeper into the concepts of onset and progression of peri-implantitis, aiming to elucidate the precise indications for employing the LbL system as a coating for implant abutments in peri-implantitis treatment. Finally, we correlate the chemical composition of the LbL system with its functionality while also addressing the challenges posed by the uncontrolled environment of the oral cavity, which ultimately restricts its clinical applicability.

## 1 Introduction

Periodontal disease has been reported to affect millions of patients worldwide according to the World Health Organization (WHO), impacting up to 1 billion of the global adult population (www.who.int/news-room/fact-sheets/detail/oral-health). Periodontitis (PD), characterized by chronic inflammation of the supporting tissues around the teeth, develops due to a complex interaction between the host and parasites, progressively compromising the integrity of the periodontal tissues.^1,2^ It is characterized by bacterial-induced inflammatory responses and the destruction of periodontal tissues, including the periodontal ligament, cement, and alveolar bone. The more severe stages of periodontitis (stage III and IV) affect over 700 million people, representing approximately 11% of the global population.^3^ Indeed, PD ranks as the sixth most prevalent chronic condition globally and is considered the leading cause of tooth loss in adults.^4,5^ Consequently, PD presents a significant public health challenge due to its high prevalence and the substantial burden caused by tooth loss and impaired chewing function, negatively impacting the quality of life.

Importantly, a history of PD poses a significant risk factor for peri-implantitis, which refers to the irreversible pathological condition occurring in tissues around dental implants.^6^ Historically, there has been a lack of consensus regarding the true prevalence of peri-implantitis, with heterogeneity in results attributed to variations in disease definitions and a lack of standard diagnosis.^7−10^ However, contrary to previous assumptions, peri-implantitis as a biological complication has been significantly underestimated for many years. It was only after the 2017 World Workshop on the Classification of Periodontal and Peri-Implant Diseases and Conditions that clinical parameters became better defined, revealing a prevalence of this condition much higher than expected.^11^ As such, recent studies have reported a troubling prevalence of peri-implantitis, ranging from 34% to 56% at the patient level within an average time frame of 2–7.8 years after prosthesis loading.^11^ These numbers underline not only the concerns for public health but also the significant economic burden caused by peri-implantitis. In view of this, the global peri-implantitis treatment market has been projected to reach a value of US$ 4.5 billion by 2032. Evidently, these projections are based on the increasing number of dental implants that have been and will be used for oral rehabilitation. The alarming peri-implantitis prevalence percentage further indicates that current clinical procedures aimed at preventing and treating peri-implantitis are insufficiently effective.^12^

The primary disease-causing factor for peri-implantitis is the biofilm accumulated on dental implant components at the tissue–oral cavity interface. Therefore, nonsurgical supportive therapy involving biofilm removal is a mandatory step at the initial stage of the peri-implantitis treatment. The treatment of peri-implantitis ranges from nonsurgical to surgical procedures, depending on the outcomes of the initial steps.^13^ Regardless of the chosen approach, the main objective of the treatment is to achieve complete resolution of the inflammation by biofilm removal and microbial agent elimination. In view of this, several methods have been described to achieve surface decontamination, such as the use of antiseptics,^14−16^ local and systemic antibiotic administration,^17,18^ and lasers and antimicrobial photodynamic therapy.^19,20^ However, the limited number of studies to support the benefits of adjunctive therapy compromise the reliability of the evidence and make the valuation of conventional treatments difficult.^21,22^

Biomaterials offer a range of possibilities for developing therapeutic interventions, from the use of polymeric films with antimicrobial effects on dental implant surfaces to the use of drug delivery systems or even using titanium modifications on implant/abutment surfaces to prevent and/or treat peri-implantitis.^23−25^ However, despite the large number of antimicrobial coating approaches discussed by literature, to date, all proposed surfaces have not reached the commercial stage.^26^ A well-designed review^26^ clearly revealed a growing number of published articles under the antimicrobial coatings field, with potential highlights on the preclinical studies. This result confirms that the stagnation in clinical application might be attributed to the complexity of the factors involved in the antimicrobial coating development, from product construction to product application. Indeed, problems in translating preclinical findings to clinical applications can be attributed in part to cytotoxicity, material behavior, drug release control, and duration of product antimicrobial activity to make it clinically viable.^26^ However, the lack of knowledge about the clinical purpose of the desired material might directly affect the success of the antimicrobial coating.

In fact, understanding the rationale behind developing a surface modification is the initial step toward successfully acquiring new materials. It is imperative, however, to consider the distinctions between preventive and treatment approaches in guiding the complexity of biomaterials. As peri-implantitis is an inflammatory condition initiated by a dysbiotic biofilm adherent to a substrate, preventive strategies are related to material development with direct antimicrobial action, through either contact killing or antifouling surfaces.^27^ In this case, the material should be resistant to pH differences caused by food consumption and withstand the mechanical action of brushing to maintain its function in the long term. From a treatment perspective, biomaterial-based coatings should be easily degraded to directly combat the infection and/or the inflammatory response, through the release of antimicrobial agents and/or substances implicated in the inhibition of osteoclastogenesis, and restore the health of peri-implant tissues in patients diagnosed with peri-implantitis.^28−31^ To this purpose, drug delivery technologies have enabled the delivery of a therapeutic to its target site, minimizing off-target accumulation and facilitating patient compliance.

Drug release technology provides a higher local drug concentration to specific sites on and around the implant, thereby offering immediate action against implant-associated infections. The basic idea behind drug release systems is to create a responsive structure, called a smart coating, on the surface capable of loading drugs and releasing them in a regulated manner over time. To this end, chemical cross-linking processes and layer-by-layer (LbL) systems^32−36^ constructed using natural and/or synthetic polymers^108^ might act as intelligent strategies for releasing the loaded drugs. Among the techniques for building smart coatings, here we will focus on LbL assembly as an appealing strategy to immediately fight against the already installed disease.^28^ LbL assembly has many advantages such as the high reproducibility of film formation in an easy, versatile, flexible and inexpensive process, nanometer control over film thickness, and a wide variety of natural and/or synthetic polymeric materials to be used as multivalent species to build up the film.^37−39^ Basically, the LbL method involves the alternating adsorption of complementary multivalent species on a substrate through electrostatic interactions, hydrogen bonding, or other secondary interactions, which will work as a structure for drug incorporation.^28,39^

While research on LbL technology has expanded in recent years, the challenge of translating it into commercial products may stem from a lack of understanding related to the system limitations, coating purpose, and the meaning of disease stage for its application. To the best of our knowledge, this is the first study that breaks down the barriers of the LbL system description and provides detailed chemical and biological information to clarify its strict application in terms of physical site and purpose as antimicrobial coatings. Likewise, we give a deeper overview of the onset and progression concepts of the disease to clarify the indications for the LbL system as a coating for implant abutments aimed at the treatment of peri-implantitis. Finally, we relate the chemical nature of the LbL system to its functionality and discuss the oral cavity as an uncontrolled environment that limits its clinical applicability.

## 2 Understanding Peri-implantitis

**Figure 1:**
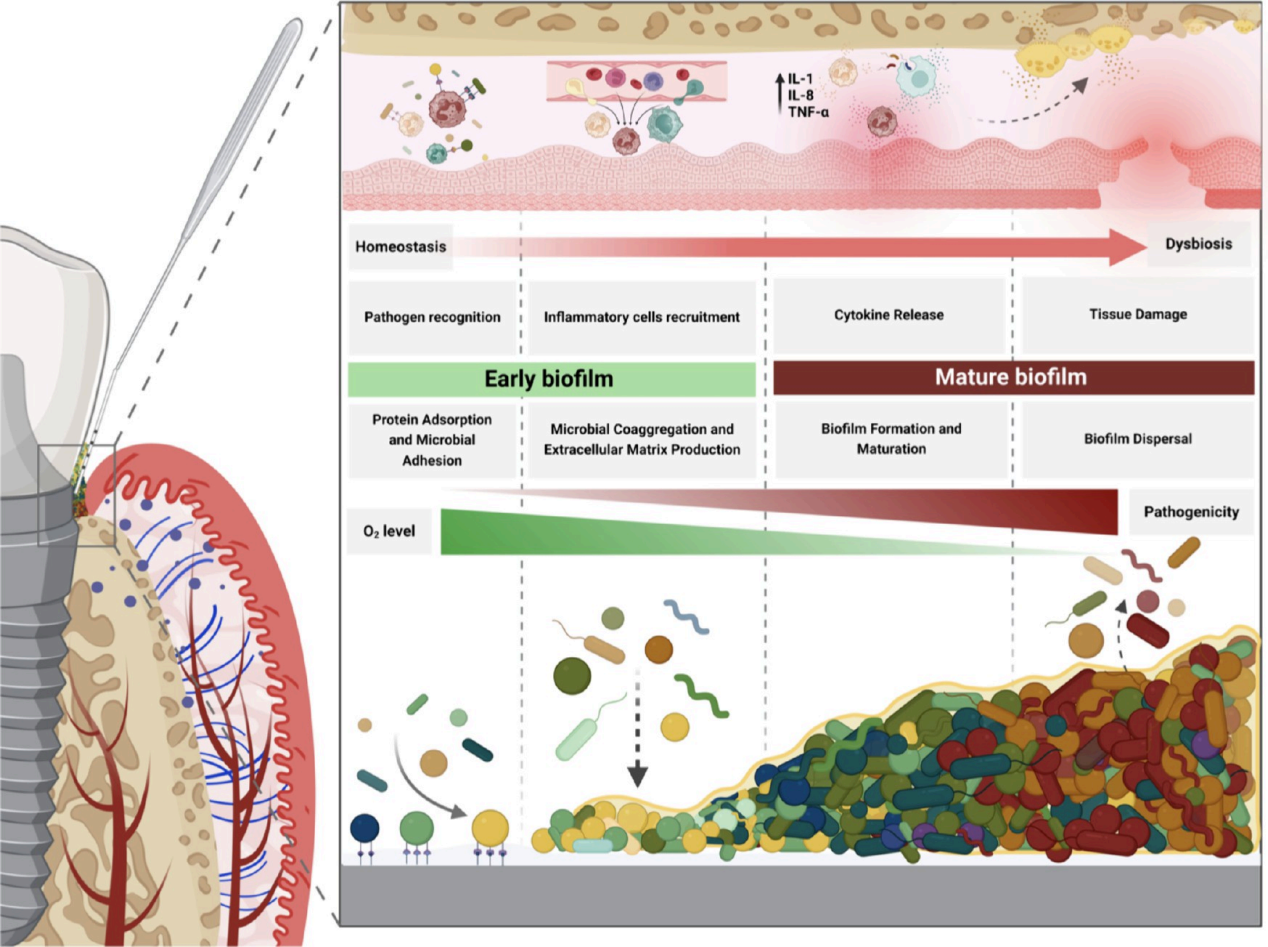
Schematic representation depicting the process of biofilm formation and its interplay with the inflammatory cascade. Upon introduction of implants or abutments into the oral environment, they are exposed to protein-rich fluids such as blood plasma and saliva, facilitating the adherence of a diverse array of proteins onto their surfaces. These proteins serve as binding sites for bacterial adhesion, thereby facilitating bacterial adherence. Early colonizers initially adhere to the surface, fostering conditions conducive to microbial coaggregation and subsequently enabling the incorporation of late colonizers. Biofilm maturation involves the secretion of an extracellular matrix, providing protection against immune cells and antimicrobial agents. Within these structures, microbial cells have the capability to detach and migrate to new sites, a phenomenon termed biofilm dispersal. Simultaneously, resident cells in the peri-implant tissue recognize pathogens, triggering an inflammatory response. This cascade entails the recruitment of additional immune cells to the site of infection and the release of pro-inflammatory cytokines such as IL-1, IL-8, and TNF-α. This heightened inflammatory state not only induces damage to soft tissue but also contributes to bone resorption. Created with BioRender.com (License Number: ZS26S7K65G).

According to the 2017 World Workshop on the Classification of Periodontal and Peri-Implant Diseases and Conditions, peri-implantitis is a pathological condition characterized by inflammation around dental implants with subsequent loss of supporting bone.^6,40^ In the clinical setting, peri-implant mucositis is assumed to precede peri-implantitis, signaling the initial state of the inflammatory process. The onset of peri-implant mucositis exhibits clinical signs of red aspects around the implant with swollen soft tissues and the presence of bleeding and/or suppuration on probing, yet without bone loss.^40^ As a more severe inflammatory condition, peri-implantitis is influenced and regulated by the immune response. While the inflammatory reaction contributes to bone resorption around the implant, the dysbiotic biofilm adherent to the surface consistently triggers the innate response^10,41−45^ (Figure 1).

Once the biofilm is well established onto the dental implant components, the immune system responds to it leading to immunoinflammatory mediators.^41,42^ The immune system mobilizes macrophages, neutrophils, and T- and B-cells, promoting the release of pro-inflammatory cytokines interleukin-1 (IL-1) and tumor necrosis factor-alpha (TNF-α), anti-inflammatory cytokines, e.g., interleukin-10 (IL-10), and chemokines, e.g., interleukin-8 (IL-8), that lead to osteolysis and inflammatory tissue damage.^41,42,46^ The lesions at peri-implantitis sites harbor larger proportions of polymorphonuclear leukocytes (PMN) and M1 macrophages phenotypes when compared to periodontitis.^6,47^ Macrophage polarization to M1 indicates a strong pro-inflammatory response with high expression of pro-inflammatory products, such as IL-1β, IL-6, IL-12, and TNF. The secretion of such cytokines activates osteoclast precursors and contributes to the bone resorption. The higher expression of M1 macrophages may be associated with the faster progression of peri-implantitis compared to periodontitis.^47−49^

In fact, some individuals exhibit an exacerbated response to the inflammatory process, making them more susceptible to developing peri-implant diseases.^41,46,50,51^ Some conditions such as uncontrolled diabetes mellitus, autoimmune disorders, genetic factors, smoking and alcohol consumption, and some treatments such as bisphosphonate therapy, head and neck radiotherapy, chemotherapy, antibiotics use, and anti-inflammatory medications are known to alter the microbiome and hence the inflammatory response, which makes these individuals more susceptible to inflammatory conditions.^46,52,53^

Peri-implantitis is a relatively recent condition, presenting numerous challenges in comprehending its risk factors that influence disease progression and its impact on systemic diseases.^54^ For instance, existing literature confirms poor oral hygiene and a history of periodontitis as significant risk factors for peri-implantitis.^6,52,55,56^ Despite longitudinal studies affirming the influence of metabolic conditions like diabetes and habitual tobacco smoking on periodontitis, conclusive evidence regarding their effects on peri-implantitis remains elusive.^6,54^ However, examining the biological pathways implicated in smokers, it is apparent that similarities exist in the inflammatory responses between periodontitis and peri-implantitis, suggesting potential consequences of inflammatory products.^54^ Nicotine, in particular, not only enhances the production of pro-inflammatory cytokines by osteoblasts, such as IL-6 and TNF-α, known to contribute to bone resorption, but also plays an important role in decreasing PMN chemotaxis and in the hyperactivation of macrophages (which secrete considerable pro-inflammatory mediators), in addition to decreasing vascularization.^54,55,57^ If left untreated, this may lead to peri-implant diseases and eventual implant loss. However, it is crucial to note that the absence of evidence may stem from a lack of longitudinal studies establishing such associations and causality.

Evidently, dental implants are more prone to pathogen invasion compared to natural teeth due to several factors. Basically, in a natural tooth, cementum is a sheathlike structure covering the surface of root dentin and providing anchorage for collagen fibers in a pattern of perpendicular orientation.^58^ Furthermore, epithelial cells attach to the enamel and cementum via hemismosomes to achieve epithelial sealing around the teeth. Hemidesmosomes are a set of highly specialized epithelial attachment apparatus components responsible for providing stable and firm attachment to the extracellular matrix, intracellularly filament, and cytoskeleton.^59,60^ The tight seal at the soft tissue on the alveolar bone and the teeth surface plays a crucial role in serving as the first protective barrier to prevent bacterial infiltration.^61,62^ Unlike a tooth, the implant lacks structures such as root cementum and periodontal ligament responsible for connecting the soft tissues to the tooth through dentoalveolar and dentogingival fibers.^7,63,64^ Even after proper healing, the tissue around dental implants experiences reduced blood flow due to poor vascularity and a deeper sulcus, allowing bacteria to penetrate deeper due to the absence of collagen fibers (known as Sharpey’s fibers) inserted into the implant structure. The absence of such structures affects the direction of the collagen fibers. Histologically, collagen fibers are oriented parallel or circumferentially to the region of the abutment surfaces, resulting in a weaker connective interface,^57,63^ lack of soft tissue resistance against the inflammatory response, and hence rapid disease progression.

In view of the differences in pathology and histological organization around percutaneous natural teeth and dental implants, it is straightforward to state that peri-implantitis treatment poses a significant challenge to clinicians. In this sense, an important question can be raised to conceptualize the outcome of treating peri-implantitis. From a clinical standpoint, the diagnosis of peri-implantitis requires clinical signs such as presence of bleeding and/or suppuration on gentle probing, increased probing depth compared to previous examinations, and presence of bone loss beyond crestal bone level changes resulting from initial bone remodeling.^65^ During the treatment of peri-implantitis, it is not expected to recover the lost bone. According to the European Federation of Periodontology (EFP) S3 level clinical practice guideline, 2023,^22^ reconstructive procedures can be used in the management of osseous defects, as a sequential stage of surgical treatment of peri-implantitis. Surgical approaches can be employed in the sites with persisting signs of pathology after nonsurgical therapy, to provide access to the implant surface and achieve the resolution of the inflammatory lesion. Clinically, the end points of successful surgical therapy of peri-implantitis can be translated by ≤1 point of bleeding on probing, absence of suppuration on probing, probing depth ≤5 mm, and absence of progressive bone loss compared to pretreatment bone levels to verify disease resolution. Although the EPF recommends the surgical management of osseous defects in peri-implantitis patients, through the access flap with or without reconstructive procedures, no evidence demonstrating superiority of any specific surgical technique has been identified.^22^ In short, researchers in the fields of dentistry, (material) engineering, and tissue regeneration are confronted with a substantial clinical problem to address with innovative treatment approaches. Therefore, understanding and delineating the initiation and progression of the disease are pivotal initial steps toward devising effective treatment strategies to be discussed below for this biological complication.

## 3 Why Do We Still Need New Strategies to Treat Peri-implantitis?

While the progressive tissue destruction caused by peri-implantitis stems from the host’s inflammatory response, it is the flora of pathogenic microorganisms within the dysbiotic biofilm that evokes the initiation of this inflammation.^66^ According to the EFP clinical practice guideline, the initial treatment approach for peri-implantitis should prioritize nonsurgical methods that focus on biofilm removal and implant surface decontamination to control peri-implant biofilms and inflammation.^66^ Ideally, decontaminating the implant surface should be performed through supramarginal and submarginal instrumentation. For the latter, EFP guidelines recommend performing nonsurgical supra- and submarginal instrumentation with curets and/or (ultra)sonic devices as the basic control intervention methods.^67−69^ However, there is a risk of damaging the implant surface while attempting to remove the biofilm.^13,70^*In vitro* studies have demonstrated that released titanium particles following implant surface damage have the potential to trigger severe inflammatory responses via macrophages and osteoclasts^71^ and to modulate the peri-implant biofilm to a dysbiotic state.^72^ To minimize this risk, curets are now being crafted from softer materials like plastic or carbon fiber instead of stainless steel.^73^ Nevertheless, regardless of material type, curets may not completely eliminate adhered biofilm since they cannot reach the areas between the threads of the implants.^73^ Moreover, given the complexity of an already established peri-implant dysbiotic biofilm, ensuring its successful disorganization in areas with limited access further complicates treatment.^71^

Surgical strategies may be considered when nonsurgical treatments fail or in cases of moderate or severe peri-implantitis, offering greater access to peri-implant regions.^6,74^ According to evidence-based recommendation, surgical intervention is indicated for patients diagnosed with peri-implantitis, for whom end points of nonsurgical therapy (i.e., probing depth ≤5 mm and ≤1 point of bleeding on probing) have not been achieved.^75^ The goal of surgical therapy is to provide direct access, through flap elevation, to facilitate procedures required for implant surface decontamination and achieve resolution of the inflammatory process.^74^ Although the literature has presented the use of air-polishing, Er:YAG laser, chlorhexidine or photodynamic therapy, or even adjunctive local antibiotics, for implant surface decontamination, the EFP does not recommend these treatments due to lack of evidence on efficacy.^76,77^ Despite literature suggesting that chemical agents can enhance disinfection by reaching mechanically inaccessible niches, in clinical practice, local antibiotics are typically administered via irrigation, leading to easy dissolution of the applied antibiotic and thus limited therapeutic effect.^78^ Furthermore, the resident microbiota and its matrix can impede the access of local antimicrobials, preventing their penetration into deeper layers of the biofilm.^78^

The prescription of antibiotics for systemic treatment along with (non)surgical therapies is still a concern and has not been recommended by the EFP.^76,79,80^ In comparison to planktonic bacteria, those bacteria within biofilms exhibit greater tolerance to antimicrobial agents, rendering systemic antibiotics less effective as adjuncts.^81^ A recent scoping review further concluded that, although there is insufficient data to support evidence-based antibiotic protocols for peri-implantitis, systemic metronidazole adjunct to mechanical debridement improved the clinical outcomes of nonsurgical treatment.^80^ Overall, research on systemic antibiotics for peri-implantitis treatment is scarce and inconclusive, and due to the heterogeneous administration protocols reported in the literature, there is no consensus on any effective antibiotic protocol for treating peri-implantitis.^82^ When indicated, a careful risk/benefit assessment should be conducted to evaluate the potential for adverse events (e.g., allergic reactions) and antibiotic resistance.^82^ While it can be argued that all available means should be incorporated into the treatment plan given the difficulty of achieving successful peri-implantitis treatment, overall antimicrobial agents should not be used empirically due to the overgrowth of difficult-to-eradicate opportunistic pathogens.^18^

In fact, the high prevalence of peri-implantitis highlights that no clinical procedure can yet be considered effective in treating this pathological condition. Moreover, peri-implantitis might not only lead to implant loss but also poses a risk of pathogenic bacteria from the infected oral site reaching the bloodstream, causing significant morbidity and generating considerable healthcare costs. Recent work has highlighted the potential links between peri-implant health and systemic inflammation, including uncontrolled diabetes mellitus, psychological stress, cardiovascular disease, obesity, and even recent infectious diseases that caused severe acute respiratory syndrome caused by SARS-CoV-2.^83^

Similar to periodontitis, chronic inflammation around dental implants harbors pathogenic bacteria and potential pro-inflammatory cytokines that can influence other systemic inflammatory conditions, such as cardiovascular diseases. Multiple studies have shown that peri-implantitis and cardiovascular diseases share common inflammatory pathways, potentially leading to an increased risk of cardiovascular events in individuals with untreated peri-implantitis.^54,83−85^ Diabetes is another possible disease that could be influenced by peri-implantitis due to the fact that this oral disease could bring a hyperinflammatory state, which could exacerbate insulin resistance, thus impairing glycemic control and the reparative capacity of the body.^54,83^ Although there is insufficient evidence to confirm such relationships mentioned above, the consequences of peri-implantitis progression may extend beyond implant loss, underscoring the need for strategies to stabilize the disease in the short term.

## 4 Developing a Drug Delivery System Using Layer-by-Layer (LbL) Technology to Treat Peri-implantitis

**Figure 2:**
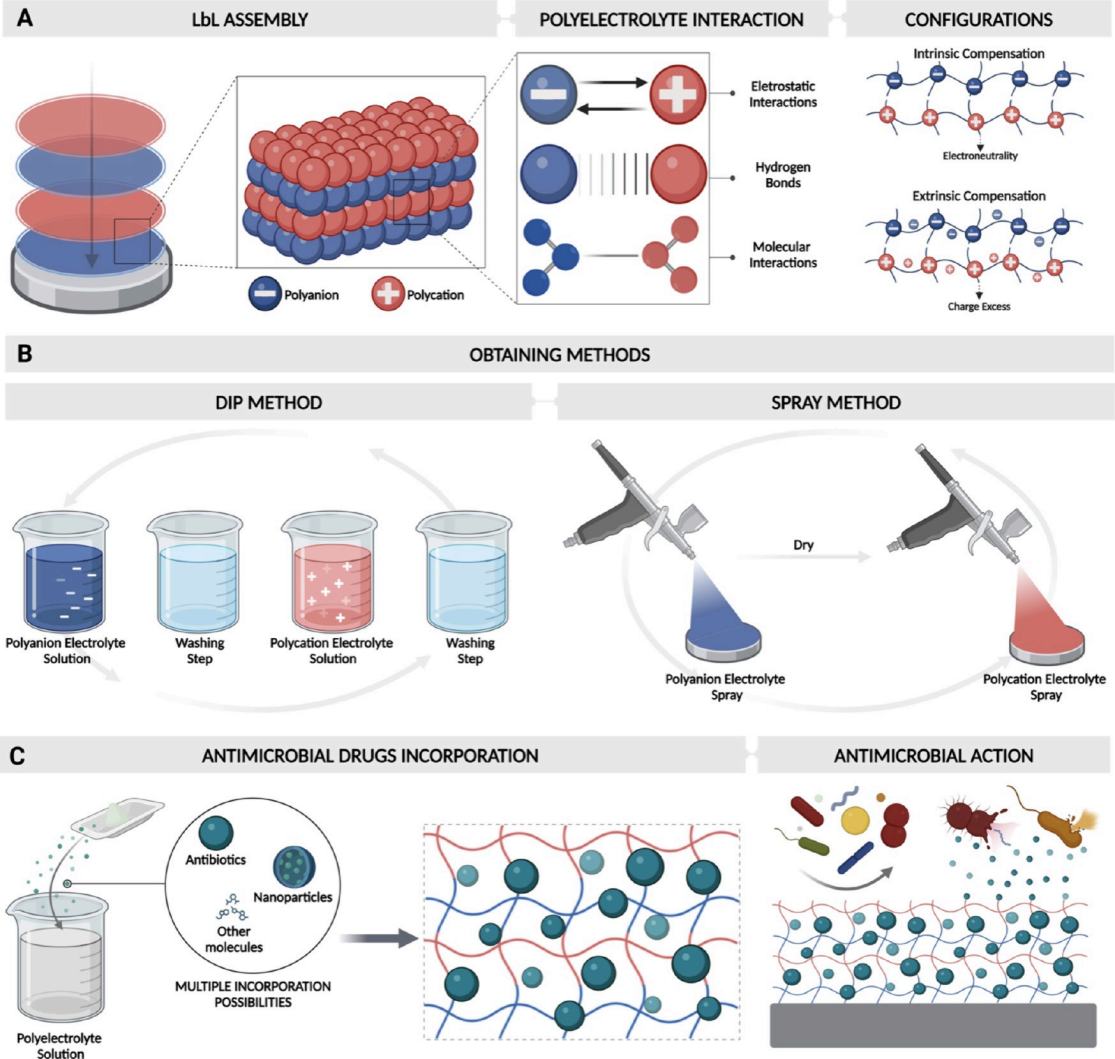
Schematic representation illustrating the layer-by-layer (LbL) assembly technique, focusing on the electrostatic interactions between polyelectrolytes. Polyelectrolyte interactions encompass electrostatic forces, hydrogen bonds, and molecular interactions. The configurations of the LbL assembly include intrinsic compensation, maintaining electroneutrality, and extrinsic compensation, resulting in excess charge. LbL assembly methods encompass the dip method, involving alternating deposition of a polycation and a polyanion electrolyte with washing steps, as well as the spray method. Additionally, the LbL system can be enriched with antimicrobial drugs, molecules, or nanoparticles, facilitating their controlled release to exert antimicrobial effects as required. Created with BioRender.com (License Number: OQ26S7K6AI).

LbL assembly is a promising avenue in the field of peri-implantitis management due to its applicability with a variety of substances, in addition to being an easy, versatile, and flexible process for multilayer formation to act directly in the affected site.^37,38^ Given that peri-implantitis is already established and requires prompt treatment, it is crucial to consider therapeutic interventions that address the inflammatory and infectious nature of the disease. Controlled and local administration of antimicrobial agents could help to reduce the local infection and subsequent inflammation, while substances targeting cells involved in bone resorption could help halt the progression of tissue loss resulting from peri-implantitis. In both cases, a drug delivery system capable of releasing therapeutic agents directly at the target site, such as via abutments, is appealing. LbL assembly offers a potential solution by serving as a biomaterial-based surface modification technology that can create a matrix for drug delivery (Figure 2A–C).

In the 20th century, Langmuir–Blodgett (LB) balances were frequently employed to deposit amphiphilic molecules at the liquid/vapor interfaces in order to manufacture films.^86,87^ Although it was initially used only on flat surfaces, this technology made it possible to create self-assembled films with low mechanical and thermal stability. In 1960, Iler introduced LbL assembly as an alternative to overcome the drawbacks of LB films, self-assembling multilayers into charged colloidal particles.^88^ Since then, LbL assembly has been applied in various fields of science and technology, including the healthcare sector, such as implants,^33,89−91^ wound healing dressings, and tissue engineering.

Basically, the deposition method for LbL assembly via electrostatic interactions involves an alternating sequence of immersing a substrate in anionic and cationic polyelectrolyte solutions, followed by a washing step between each solution exchange to ensure the complete removal of weakly adsorbed polyelectrolytes. Any deviation from this sequence may lead to nonuniform adsorption of polyelectrolytes, which can compromise the quality of the manufactured LbL^92^ (Figure 2B). LbL coatings can be assembled using a range of chemical interactions, including hydrogen bonds, charge transfer interactions, molecular recognition, host–guest, π–π, and biospecific interactions.^93−96^ From a chemical perspective, understanding the process of structuring a multilayered coating is complex and involves several factors, including charge, pH, and ionic strength of the medium.

In this sense, chemical interactions within LbL assemblies, including covalent and noncovalent interactions, are crucial for determining the stability, functionality, and drug-release behavior of these films.^93−97^ Covalent bonds, formed through the sharing of electron pairs, provide robustness and long-term stability, essential for sustained drug delivery, often enhanced through cross-linking methods like click chemistry or disulfide bond formation.^97−100^ Conversely, noncovalent interactions, such as electrostatic forces, hydrogen bonding, and van der Waals forces, drive the dynamic assembly and disassembly of LbL structures, enabling responsive and controlled drug delivery by facilitating the sequential buildup of layers with precise control over film thickness and composition.^97−100^ These interactions might be introduced during the assembly process by using reactive functional groups on the polymers or molecules that form the layers.^101^ For instance, covalent cross-linking can be employed to enhance the mechanical strength and stability of the LbL films, making them more resistant to environmental conditions such as changes in pH or ionic strength.^101^ Cross-linking can be achieved through various chemical reactions, such as click chemistry, “Schiff” base formation, or disulfide bond formation.^97−100^ These covalent bonds provide long-term stability, which is crucial for applications in which the film must remain intact for extended periods, such as in sustained drug delivery systems.

On the other hand, noncovalent interactions are the primary driving forces in the formation of LbL films.^97−100^ These interactions are generally weaker than covalent bonds but are essential for the dynamic assembly and disassembly of LbL structures.^97−100^ For example, electrostatic interactions are the most commonly utilized forces in LbL assembly, where alternating layers of positively and negatively charged polyelectrolytes are deposited.^101^ The electrostatic attraction between oppositely charged layers enables the sequential buildup of the film, allowing for precise control over the film thickness and composition.^101^

A LbL coating discloses two possible configurations: (i) adsorption in which the charge balance results in electroneutrality, called intrinsic compensation, and (ii) adsorption that results in an excess net positive or negative charge, arising from the adsorption of polycations and polyanions, respectively, called extrinsic compensation^97,98^ (Figure 2A). The multilayer system becomes unstable due to the excess charges, since the balance will be altered in favor of stabilizing these loads. Zeta potential behavior often analyzes these measurements.^99,100,102^

The ionic strength of polyelectrolyte solutions plays a crucial role in forming homogeneous layers during LbL coating buildup, and it is imperative to consider the characteristics and concentration of the ion(s). The presence of small ions, also known as kosmotropic ions, results in thin films with low hydration and roughness due to their low polarization and weak binding with the multilayer systems. On the other hand, chaotropic ions, which have a higher degree of polarization, interact more with the multilayers, leading to changes in the conformation of the polyelectrolytes and affecting the thickness of the multilayers.^103,104^ The pH of the medium also has a significant impact on the formation of multilayers, and it can alter the degree of ionization of the polyelectrolytes.^105^ Consequently, the pH modifies the organization of the multilayers.

In short, LbL assembly reveals overriding advantages related to the low cost, versatility, and simplicity of obtaining well-defined thickness, composition, and functionality materials. The low cost is related mainly to the simplicity of the technique. LbL coatings can be assembled at room temperature without pressure, which means that this technique will not compromise the integrity of reagents, such as proteins or drugs. The versatility of LbL assembly as a coating is due to its ability to vary in composition and its possibility to create defined sequences of layers.^106^ With regard to the functionality, LbL assembly has found applications within the biomedical field. In this sense, the multilayers can be purposely prepared to admit antimicrobial action or can be constructed to receive the target drug. In this perspective, the literature brings antimicrobial polyelectrolytes to be applied as a target layer during the LbL system construction.^107,108^ The explanation of the antimicrobial properties of specific polyelectrolytes is related to their hydrophobicity.

The antimicrobial capacity of hydrophobic polyelectrolytes has long been exploited as a replacement for conventional antibiotics.^109,110^ The antimicrobial effects of the polyelectrolytes are related to the nature of hydrophobic groups, polyelectrolyte composition, and length for a series of antimicrobial block polyelectrolytes.^111^ Interestingly, although cationic groups also contribute to the antimicrobial activity of the material, the hydrophobicity within the polyelectrolytes is the driving component for lipid membrane binding and pore formation which in turn causes cell death through a membrane-disruption mechanism.^112,113^ For instance, the use of poly(ethylenimine) (PEI) as a strong cationic polyelectrolyte provides inherent bactericidal activity via its cationic groups.^114,115^ These bind to phospholipids in cell membranes through electrostatic interactions, leading to membrane ruptures.

The bactericidal activity of modified PEIs was further enhanced by the addition of hydrophobic alkyl groups and a more positive charge density. It was found that linear *N*,*N*-dodecyl-ethyl poly(ethylenimine) and poly(acrylic acid) LbL coatings demonstrate strong antiviral activity against influenza A/WSN (H1N1) and bactericidal activity against *Staphylococcus aureus* and *Escherichia coli*.^116^ The balance between cationic groups and hydrophobic side chains on different sides of overall polyelectrolytes has been recognized for disrupting bacterial cell membranes and causing membrane leakage and eventually cell death.^117,118^ From this knowledge, the literature suggests a correct balance between hydrophobicity and hydrophilicity properties within biomaterial polyelectrolytes to achieve an antimicrobial interaction with bacterial membranes without causing cytotoxicity or cell repeal.

**Figure 3:**
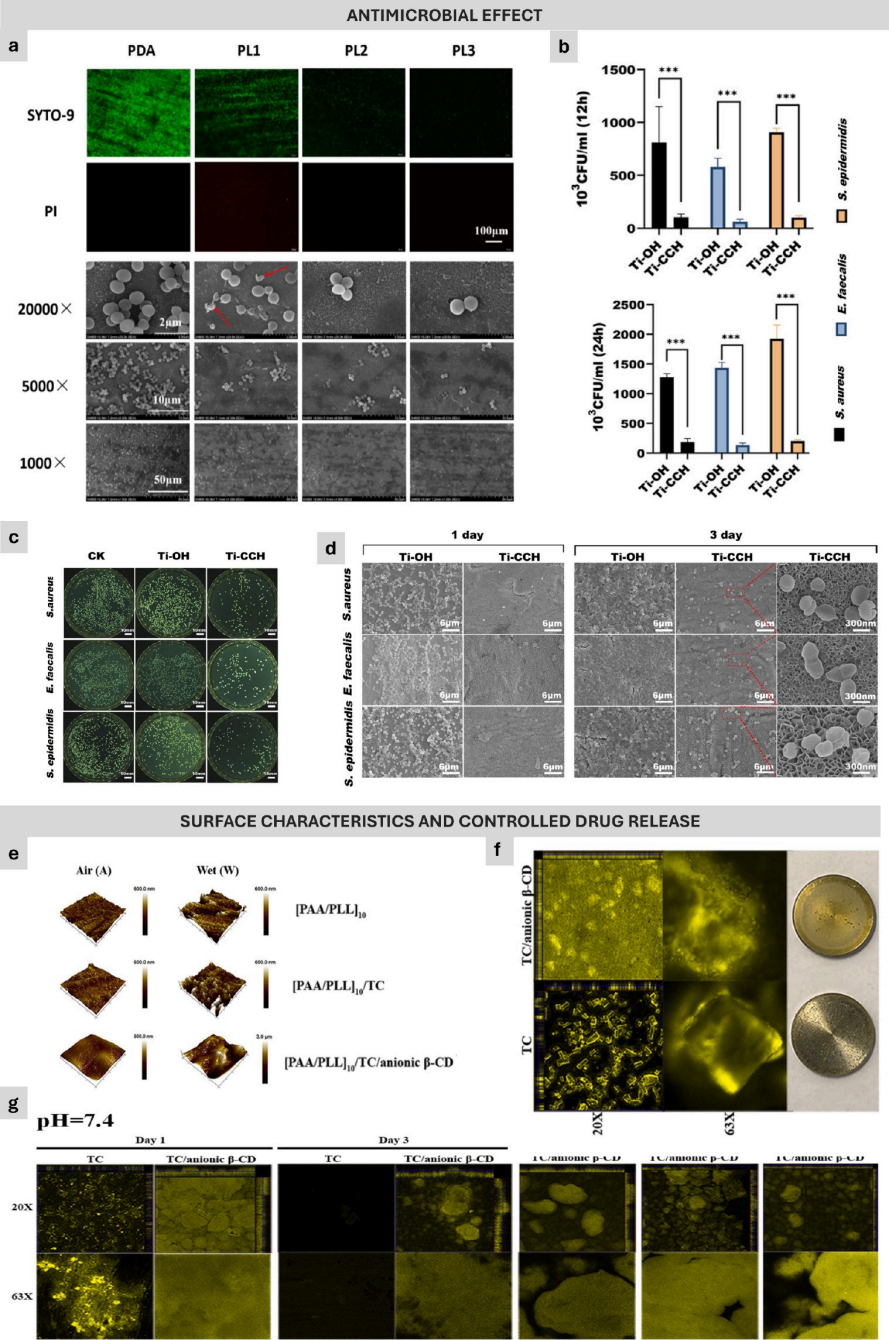
Illustration presenting diverse results from previous studies, focusing on surface characteristics and controlled drug release. The top section of the figure shows the antimicrobial effects of the layer-by-layer systems produced. Part a highlights that ε-polylysine and gum Arabic multilayer composite films on titanium substrates promoted long-term antibacterial properties against both Gram-positive *Staphylococcus aureus* (*S. aureus*) and Gram-negative *Escherichia coli* (*E. coli*). Additionally, parts b and c illustrate that quaternary ammonium carboxymethyl chitosan exhibited antibacterial action against three different bacterial strains within 12 and 24 h of testing, resulting in reduced colony-forming units and lower colonization, as depicted by scanning electron microscopy in (d). In the bottom section, the surface morphology and drug release behavior are depicted. Part e showcases the surface morphology of multilayers comprising poly(acrylic acid) (PAA) and poly(l-lysine) (PLL) integrated with (f) tetracycline on titanium surfaces, demonstrating controlled drug release facilitated by anionic beta-cyclodextrin (g). Part a has been reprinted and adapted with permission from Elsevier (License Number: 5780350302603). Parts b–d are also reprinted and adapted with permission from Elsevier (License Number: 5780370420377). Additionally, parts e–g have also been reprinted and adapted with permission from Elsevier (License Number: 5503210237348).

In order to enhance the interaction between film and substrate and optimize the antibacterial properties of titanium surfaces, another study combined ε-polylysine (ε-PL), a typical cationic antimicrobial peptide, with arabic gum, a polysaccharide, for LbL construction. They used polydopamine to covalently graft ε-polylysine onto anodized titanium. The antimicrobial capacity of this system was attributed to the strong electrostatic interaction between the negatively charged acidic phospholipid of bacterial cell membranes and the positive charge from ε-PL. The wizened and ruptured morphology of *S. aureus* and *E. coli* on coated Ti surfaces suggested that ε-PL effectively killed bacteria by destroying their cell membranes. However, the limited amount of ε-PL covalently conjugated on the surface affected the antibacterial efficiency. Interestingly, decreased bacterial adhesion was found on the surface along with increased layers of ε-PL and GA, suggesting that these reagents, bound by electrostatic adsorption, might dissolve into the solution during the bacterial incubation process^119^ (Figure 3A–B).

Despite the apparent advantages provided by the LbL assembly, it is important to highlight the inherent limitations of LbL assembly. The most common method for LbL assembly is the sequential dipping method, which does not allow for substantial control over the orientation of the adsorbed components in the multilayers.^38^ Basically, different polyelectrolyte solutions are used to fill individual containers, and a specimen is immersed sequentially/repetitively in these solutions, with intermediate washing steps, until the desired number of layers is achieved.^120^ Although the LbL process is time-consuming due to the time required to reach equilibrium adsorption for each coating step, this system allows a sustained drug release overtime. Another way to buildup the LbL system is by using sequential spraying of the specimens instead of dipping. This method encompasses the advantage of nanometer control over film thickness without the disadvantages mentioned above. Moreover, spray-based LbL assembly is faster and smoother than dipping and can even be performed without a rinsing step. However, spray-based LbL assembly can lead to vastly rapid release of an incorporated drug^121^ (Figure 2B).

### 4.1 LbL Self-Assembly System as a Feasibility Tool for Drug Release

The LbL system as a thin film can also be designed in the field of biomedical science to work on drug delivery processes. Importantly, there are specific factors related to the effective incorporation of the drug and subsequent drug release by the LbL system: First, the nontoxicity property of the target drugs and the film material should be confirmed before preparing the LbL system.^122,123^ Second, the thin film-carrying drugs should disclose both chemical and physical stability to favor drug incorporation and its release.^124,125^ Third, drug release should occur in the target site. Fourth, the system should ensure a sustained release instead of a burst release and ensure a safe dose concentration at the target site.

This control can be achieved by managing the degradation rate of the LbL system, depending on the drug property to be incorporated, and considering the material and the assembled method selected for LbL preparation. In this sense, a variety of interactions and cross-linking chemistry can be employed for loading drugs onto prefabricated multilayers: overall drugs can be directly loaded onto prefabricated LbL; the layers can be built using drugs as building blocks; and finally, overall drugs can be also modified with cargoes, for protecting the drug by a shell of a stable chemical structure and preventing undesirable decomposition.^123,126^ Contrary to the concepts discussed in the present Review, a recent study developed a quaternary ammonium carboxymethyl chitosan, collagen, and hydroxyapatite multilayer coating via the LbL technique by polymerizing dopamine to prevent infections on implant surfaces. Importantly, although the objective of this study was to use a naturally fragile and degradable LbL system focusing on prevention for implants, the authors also considered the likelihood of disease after implantation, different from peri-implantitis. To achieve antimicrobial properties, the authors used 1-ethyl-3-[3-(dimethylamino)propyl]carbodiimide hydrochloride (EDC) in the presence of *N*-hydroxysulfosuccinimide (sulfo-NHS) to convert carboxyl groups to amine-reactive sulfo-NHS esters during the construction of the multilayers. EDC/sulfo-NHS were used as cross-linkers to extend the contact-killing properties and the release-killing over time^127^ (Figure 3C–D).

With the possibility of using LbL coatings as a carrier matrix for target biomolecules such as antimicrobial substances or those that act directly on inflammatory cells, it offers enormous potential as a drug delivery system. For instance, in a recent study, silver nanoparticles (AgNPs) were incorporated into LbL coatings based on oppositely charged amino cellulose (AM) and acylase. The resulting hybrid system showed an improved antibacterial and antibiofilm effect, while decreasing cytotoxicity.^128^ The beauty of the LbL assembly lies in the possibility of using it to produce a hybrid system that can enhance the efficacy of existing drugs. The capacity of the LbL system to release incorporated drugs occurs because the multilayered structures are naturally multiresponsive as a consequence of interactions among the layers, which means that they answer to different stimuli, such as temperature, pH, and humidity. It is precisely due to the capacity of the system to respond to multiple stimuli that LbL assembly displays the ability to incorporate drugs in high concentrations within a multilayer thin film and release them in a controlled or uncontrolled manner^129,130^ (Figure 2C).

In our previous work,^33,131^ we demonstrated that our LbL system changed molecular conformation upon immersion in ultrapure water and displayed a swelling behavior of the polyelectrolyte matrix. Our outcomes were also categorical in showing that when samples were kept in wet conditions, the LbL system with drug incorporated onto titanium discs displayed the highest roughness, which indicates that LbL-coating conformational changes contributed to the drug diffusion process through the multilayered coating. From a clinical perspective, the physiological environment of the oral cavity would act to disturb the stimuli-responsive polyelectrolyte of the system and allow the release of the incorporated drug for local and immediate action. To understand how the release process occurs and how to control the concentration of drugs released at the site, it is necessary to understand the chemical characteristics of both the system and the released agent.

To reach the biological performance of the controlled drug release, it is required that the system protects the drug, loads the drug, and releases the drug in a controlled manner for the needed time, which are all directly influenced by a drug’s molecular structure.^132^ Since the electrostatic interactions between oppositely charged ions from different layers are the most applied driving force in the LbL assembly, the same principle must be applied between polyelectrolyte layers and the target drug. An example includes antibiotics disclosing hydrophilic properties as target drugs, which are released more quickly upon exposure to the aqueous environment. This can be explained by the weak interaction between hydrophobic and hydrophilic molecules from polymers and antibiotics, respectively,^133^ which means that there is no driving force between the molecules from layers and loaders.^134,135^

To overcome this issue, researchers have studied amphiphilic inclusion complexes with overall drugs to enhance the drug capability to entrap into hydrophobic layers,^136^ increase the interaction between drug and LbL system, and ensure the controlled drug release overtime. In a recent study, the authors used amphiphilic molecule anionic beta-cyclodextrin (β-CD) to retain tetracycline, as a hydrophilic antibiotic, within the LbL-coating titanium substrate and control TC release from the multilayers up to 30 days^33^ (Figure 3E–G). The strong interaction between TC and anionic β-CD and effective loading of the complex within the LbL system enabled TC retention within the LbL coatings for a prolonged time, regardless of medium pH. Importantly, the strong antibacterial effect was observed after 48 h of incubation with more than 5 log reduction of bacterial growth in comparison to the uncoated titanium surfaces. The remaining antibacterial activity of the LbL system was confirmed even up to 30 days, with 2.8 log reduction of *S. aureus* compared with the same uncoated surfaces. Summarizing, Table 1 illustrates how LbL technology can be tailored for specific dental implant applications by leveraging different stimuli-responsive features. This table and summary provide a clear overview of how LbL technology is advancing the field of dental implants by improving their functionality and clinical outcomes.

**Table 1:** Advancements in LbL Assembly on Dental Implant Surfaces and Medical Devices, Focusing on the Use of Various Compositions and Drug Agents to Enhance Antibacterial Properties, Control Drug Release, and Promote Tissue Regeneration

author(s)	year	surface	methodology	LbL composition	drug/agent loaded	stimuli-responsive feature	key findings-results	type of study	control drug release results	conclusion
Escobar et al.	2019	titania-coated glass	polyelectrolyte multilayers with gentamicin applying the LbL technique	poly(acrylic acid) (PAA)/poly(l-lysine) (PLL)	getamicin	pH-responsive	preventing the proliferation of the *Staphylococcus aureus* strain	*in vitro*	initial burst release of 58% gentamicin in the first 6 h; sustainable release lasting up to 5 weeks.	Polyelectrolyte multilayers are effective in preventing the proliferation of the *S. aureus* strain.
Wongsuwan et al.	2020	titanium	LbL coating with minocycline-loaded niosome	polyelectrolytes and minocycline-loaded niosomes	minocycline	pH-responsive	minocycline-loaded niosome in LbL demonstrated sustained drug release and effective antibacterial action against oral pathogens	*in vitro*	prolonged minocycline release up to 7 days	Minocycline-LbL coatings are effective in dental implant applications for preventing bacterial infections.
Verza et al.	2021	titanium	LbL coating using β-cyclodextrin complex	PAA/PLL	tetracycline	pH-responsive	controlled release of antibiotic; reduces bacterial colonization	*in vitro*	sustained release for over 30 days	Long-term controlled drug release was effective for antimicrobial purposes.
Lin et al.	2022	titanium	chitosan-modified cross-linking LbL technique to form stable amido bonds with polymerization of dopamine	quaternary ammonium carboxymethyl chitosan (QCMC), collagen (COL I), and hydroxyapatite (HAP) multilayers coating	quaternary ammonium carboxymethyl chitosan	degraded under the action of collagenase I	the two-phase coating showed one prolonged antibacterial effect over a long time and a second-phase osseointegration-promotion capability	*in vitro* and *in vivo*	displayed slow continuous and long-lasting antibacterial activity for more than 45 days	The multiphase coating provides long-lasting antibacterial protection for titanium implants.
Kitagawa et al.	2021	titanium-based alloy	LbL coating of TiO_2_ and poly(sodium 4-styrenesulfonate)	TiO_2_ nanoparticles and poly(sodium 4-styrenesulfonate)	Ti dioxide (TiO_2_) nanoparticles	not applicable	targeted drug release with temperature trigger; reduces risk of infection in dental implants	*in vitro* and *in vivo*	not applicable	LbL coating with TiO_2_ enhanced biocompatibility and integration for dental implants.
Dwivedi et al.	2018	stainless steel medical devides	LbL coating with antibacterial niosome	poly(lactic acid) (PLA)/niosomes	vancomycin	pH-responsive	enhanced antibacterial activity against *P. gingivalis*; controlled release of antibiotic	*in vitro*	controlled drug release for 15 days	Niosome-LbL coatings showed promise for antibacterial activity on orthopedic implants.

It is essential to note that the factors that are involved in the structuring of the LbL system are also responsible for the responsive nature of the bioactive compounds. The most prevalent self-defensive antibacterial LbL films are pH-responsive because they take advantage of the release of lactic and acetic acid by different bacteria, which lowers the pH of the infection’s microenvironment.^137^ Thus, pH shifts can alter the conformational state of the layers in LbL coatings, changing the dimension of the polyelectrolyte mesh and facilitating the release of drugs.^138^ A similar effect occurs when the multilayer system meets the biological environment. The presence of salts results in the rearrangement of water molecules within the polyelectrolyte layers, whose driving force is guided by an entropic effect, modifying the conformation of the polyelectrolyte present.^138^ From this perspective, the release rate does not occur exclusively through drug diffusion and platform degradation, but the phenomena involved in extrinsic compensation are also responsible for changes in the physicochemical properties of the LbL system.

### 4.2 Physical Properties of LbL Coatings for Implant Abutment Surfaces

Implant abutments are essential components that serve as bridges or extensions attached to implants to secure artificial teeth. Unlike the implant itself, which is not subject to mechanical friction during installation, the significance of abutments lies in their direct contact with surrounding soft tissue. Hence, any polyelectrolyte coating applied to abutment surfaces should exhibit sufficient strength to withstand the forces exerted during placement without being easily being removed. Furthermore, from a clinical point of view, screw-retained abutments could be easily replaced by coated ones during disease treatment.

It is widely acknowledged that surface topography significantly influences the biological response of surrounding tissues to abutments.^139^ Numerous studies have demonstrated that rough surfaces tend to accumulate more plaque,^140,141^ increasing the risk of plaque-induced inflammatory reactions in surrounding tissues compared to smoother surfaces. Therefore, abutments should ideally feature smooth surfaces, typically less than or around 0.2 μm,^140,142,143^ to facilitate mechanical cleaning and ensure peri-implant health, especially in patients at risk of peri-implantitis. Another critical surface property affecting surface protein adsorption and cell adhesion quality is wettability, with cells showing a greater propensity to adhere to hydrophilic surfaces.^144,145^ In essence, any coating-based approach to combat peri-implantitis should preserve or enhance the inherent properties of the components.

For abutments, the primary challenge lies in developing a coating that maintains the original topography—ensuring it does not interfere with smoothness and wettability—while adding antimicrobial properties without adverse effects on human cells.^26^ From a dental implant perspective, LbL technology holds promise for coating implant abutment surfaces.^101^ The multilayers assembled onto abutment surfaces allow precise control of polyelectrolytes’ vertical dispersion at the nanoscale, thereby achieving the desired roughness based on the chosen multilayer creation process.^33,131^ In other words, LbL-coating properties such as thickness, homogeneity, and internal structure can be controlled and determined by various LbL methods.^146^

With regards to the wettability, hydrophilic/hydrophibic potential of the LbL system is a key factor that influences protein adsorption, cell adhesion, and the overall interaction between coating and biological environment.^101^ Hydrophilic surfaces may promote protein adsorption and enhance cellular attachment, which can be beneficial in the tissue engineering field.^101^ Studies have shown that LbL assembly can be used to finely tune these properties by selecting appropriate materials for the layers.^33,133−135^ For instance, incorporating hydrophilic polymers such as poly(ethylene glycol) (PEG) can decrease the contact angle, thus enhancing material hydrophilicity.^33^

**Figure 4:**
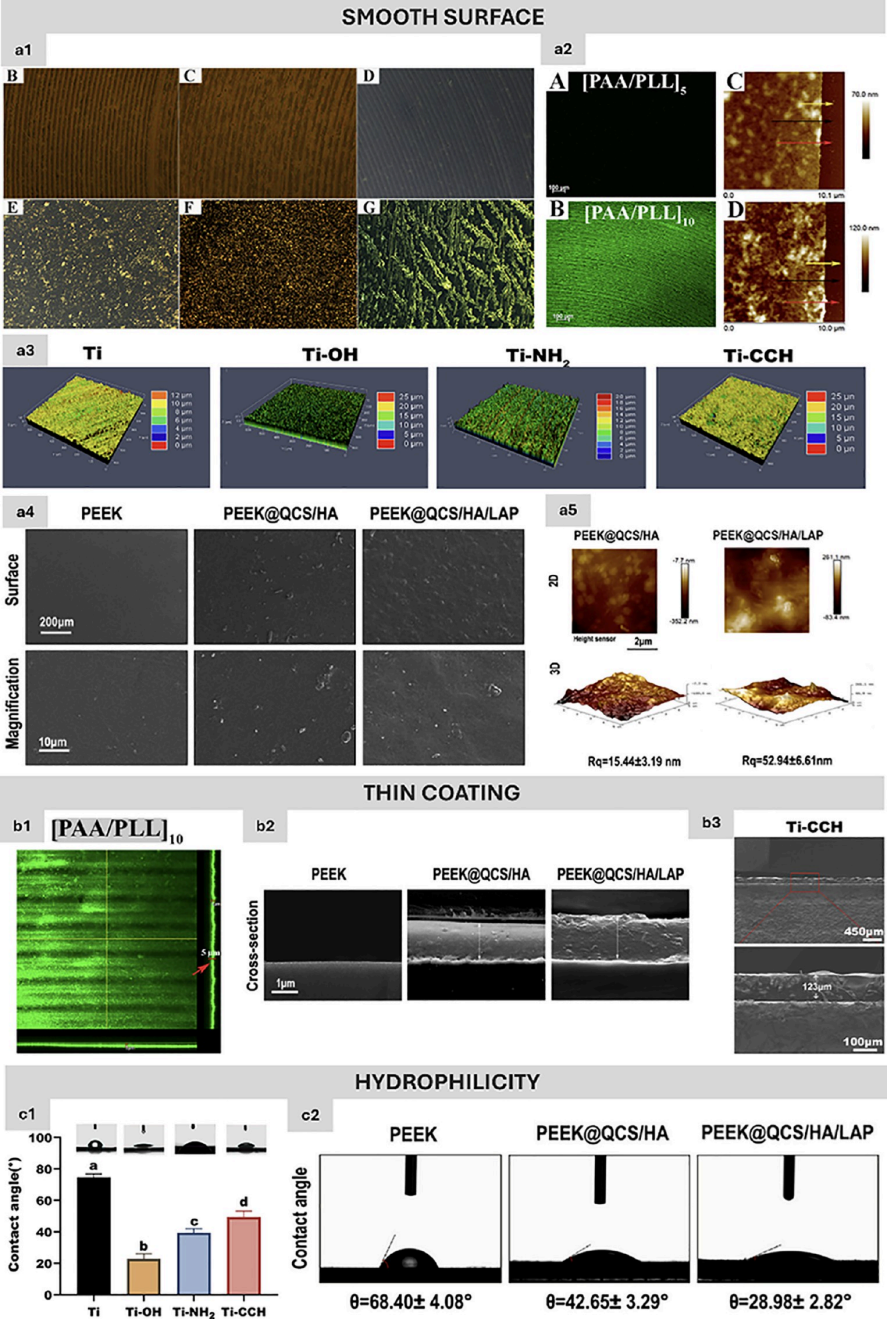
Key characteristics of ideal LbL coatings for biomedical use. (a1) Schematic representation showing the influence of temperature on the conformational changes of electrolytes and TC incorporation in [PAA/PLL]10 multilayers. (B) TC-loaded Ti implant after 4 days at 37 °C. (C) [PAA/PLL]10/TC multilayers after 4 d at 37 °C. (D) TC-loaded Ti after 24 h in PBS at 37 °C. (E) [PAA/PLL]10/TC multilayers after 24 h in PBS at 37 °C. (F) [PAA/PLL]10/TC multilayers after 24 h in acetate buffer at 37 °C. (G) [PAA/PLL]9/TC + [PAA/PLL]1 multilayers after 24 h in PBS at 37 °C. (a2) Fluorescence microscopy images confirming the successful LbL deposition on Ti discs with FITC-labeled PLL for (A) [PAA/PLL]5 and (B) [PAA/PLL]10. Quantitative analysis comparing coating thickness for (C) [PAA/PLL]5 and (D) [PAA/PLL]10, with red, black, and yellow arrows indicating measurements at various regions. (a3) Surface roughness characterization of Ti, Ti–OH, Ti–NH_2_, and Ti–CCH substrates using confocal laser scanning microscopy (CLSM), following QCMC, COL I, and QCMC/COL I/HAP multilayer coatings. (a4, a5) Surface morphology of QCS/HA/LAP-coated PEEK films, visualized through (C) SEM, while (a5) presents AFM images showing surface roughness and topography of the coatings. (b1) Confocal microscopy illustrating the uniform distribution of [PAA/PLL]10 coatings on Ti discs, with fluorescent bands highlighting the coating thickness (red arrow). (b2) SEM cross-sectional images of uncoated and QCS/HA/LAP-coated PEEK films. (b3) SEM images depicting the thickness of the QCMC/COL I/HAP multilayers on Ti–CCH surfaces. (c1) Water contact angle measurements comparing Ti, Ti–OH, Ti–NH_2_, and Ti–CCH, with synthetic QCMC, COL I, and QCMC/COL I/HAP coatings. (c2) Contact angle analysis of bare and QCS/HA/LAP-coated PEEK films, demonstrating improved hydrophilicity of the coated surfaces. Parts a1, a2, and b1 are reprinted and adapted with permission from Elsevier (License Number: 5876160692351). Partss a3, b3, and c1 are reprinted and adapted with permission from Elsevier (License Number: 5780370420377). Parts a4, a5, b2, and c1 are reprinted under the terms of the Creative Commons CC BY license.

Owing to the versatility and flexibility of LbL assembly, coating surface properties can be easily modified to enhance their biological response before they come into contact with cells and tissues^146^ (Figure 4). However, caution is warranted when considering this technology for implants themselves due to the risk of displacement or physical damage to LbL coatings during implant installation. Clearly, LbL assembly is a system based on a polyelectrolyte responsive to various conditions, suggesting its susceptibility to mechanical forces.

## 5 Key Challenges for an LbL-Based System to Fight Peri-implantitis

While the LbL system holds significant potential, it also faces several key challenges that must be addressed for its successful implementation:

- *Limited Long-Term Efficacy*. One of the primary challenges with the LBL system is ensuring its long-term efficacy in preventing peri-implantitis recurrence. The durability of the coatings and their ability to withstand the oral environment, mechanical stresses, and microbial challenges over time are crucial factors.^38,39^ Researchers are actively investigating new materials and coating techniques that offer enhanced stability and long-lasting protection against infection and/or inflammatory processes.^33,147^
- *Biocompatibility and Tissue Response*. The biocompatibility of the materials used in LbL coatings is essential for promoting tissue integration and minimizing adverse reactions.^37,148^ Ensuring that the coatings do not trigger inflammation or immune responses is crucial for the success of the implant. Researchers are exploring novel biomaterials and surface modifications to improve biocompatibility and enhance soft tissue integration.^33,147^
- *Optimal Coating Thickness and Composition*. Achieving the right balance in coating thickness and composition is another challenge. Coatings that are too thin may not provide adequate protection against bacterial infiltration, while overly thick coatings can interfere with tissue integration and healing.^38,39^ Finding the optimal combination of materials and layering techniques is an ongoing area of research.
- *Control over Drug Release*. Achieving controlled release of a drug at the targeted location for the time necessary to achieve the effect of the treatment is one of the major challenges to be addressed. The main goal of LbL-targeted drug delivery systems is to obtain high enough local concentrations of drugs through spontaneous physiological processes with low systemic exposure. Moreover, the high drug concentration incorporated into the system should not be cytotoxic to human cells and tissue surrounding the implant component.
- *Mouth as a Complex and Uncontrolled Environment*. The human mouth is a complex system constantly exposed to saliva, bacteria, enzymes, and biochemical processes related to the pH variation. The pH is influenced by diet, oral hygiene practices, medical conditions, and medications. Although saliva plays a pivotal role in maintaining the pH balance in the mouth, a consistent imbalance in oral pH can affect the molecular structure of the LbL system. Indeed, saliva is one of the most important fluids to interact with overall biomaterials in the first instance. The interaction of saliva compounds with biomaterials may cause chemical–physical–biological alterations in biomaterials.^149,150^ In fact, in the present Review, we have underlied the LbL system to peri-implantitis treatment due to its ability to release the target drug over time. LbL biodegradation governs the process of drug release, and the release profile of the drugs depends upon the nature of the delivery system. However, in addition to the chemical factors, physical forces related to the chewing, swallowing, or brushing can directly affect the degradation process and accelerate the drug release.^151^ Definitely, LbL may be defined as a limited system by the fact that it can release only one dose at a period of time. However, within the oral cavity, its particular physiological conditions can be considered confounding factors, promoting stimuli for the unexpected degradation of the biomaterial, reducing the shell life of the system and compromising the applicability of the coating.
- *Clinical Translation and Standardization*. Moving from laboratory research to clinical application presents its own set of challenges.^26,38,39^ Ensuring that LbL coatings can be easily applied during implant rehabilitation, are cost-effective, and have predictable outcomes in diverse patient populations is crucial. Standardizing coating techniques and protocols across different dental practices and implant systems is essential for widespread adoption.
- *Regulatory Approval and Safety*. Before LbL coatings can be used clinically, they must undergo rigorous testing for safety and efficacy.^39,40^ Obtaining regulatory approval from health authorities requires extensive preclinical and clinical studies to demonstrate the coatings’ benefits and safety profiles. Meeting these regulatory standards adds to the challenges of bringing LbL approaches to the market. A major concern is the new MDR (medical device regulation) for which any adjustment to an existing/approved product requires rigorous testing and documentation and high costs for regulatory approval (https://commit-global.com/how-does-the-eu-mdr-affect-the-translation-process/).

Despite these challenges, the LbL system offers exciting possibilities for improving the success rates of dental implants and reducing the incidence of peri-implantitis.^33,38,39,148,148^ Ongoing research and technological advancements are gradually addressing these obstacles, paving the way for the development of innovative coating materials that enhance the longevity and performance of dental implants.

Toward the end of this Review, it is essential to address the current progress in translating Layer-by-Layer (LbL) antimicrobial coatings for dental implants from the laboratory to clinical application. This section covers the stage of development of LbL coatings, the preclinical testing models used, and the prospects for clinical translation.

## 6 Current Progress in Translating LbL Antimicrobial Coatings for Implant Abutment Surfaces

**Figure 5:**
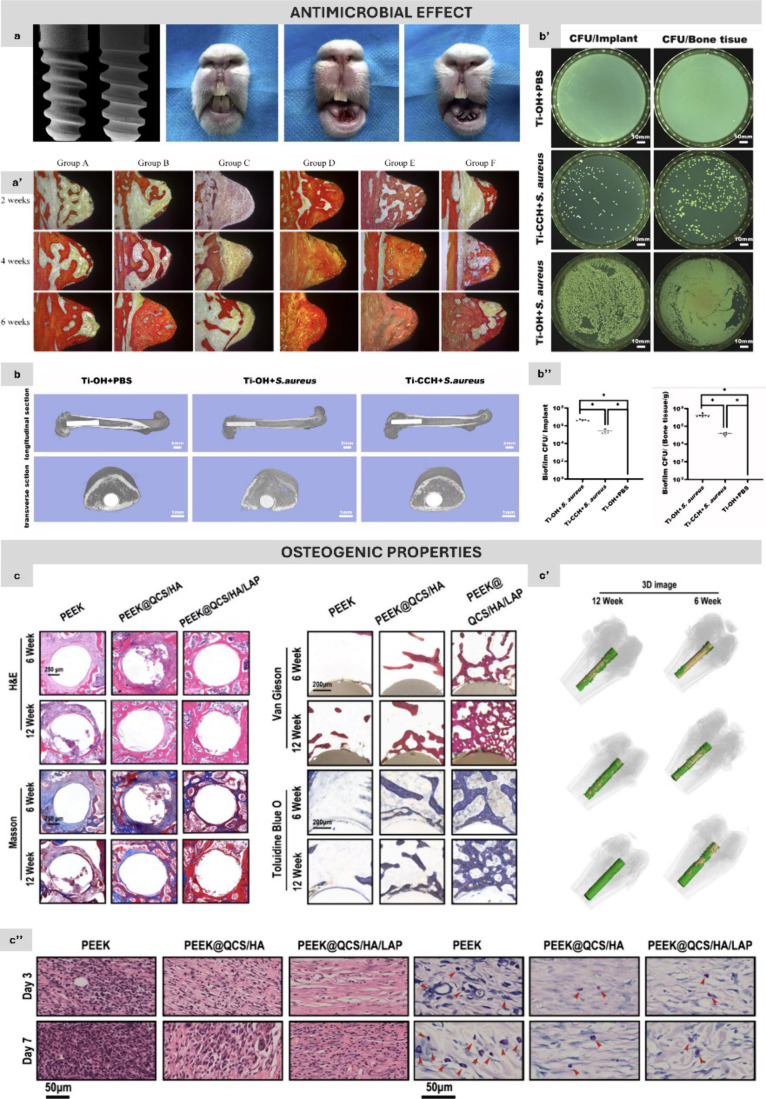
*In vivo* osseointegration and antibacterial performance of layer-by-layer systems. (a) SEM images of SLA (left) and Ti (PLL/CA-3.0) (right) implants, alongside photographs of the implantation procedure, showing the anterior teeth region of New Zealand white rabbits, the alveolar socket postextraction, and after implant insertion. (a′) Histomorphometric analysis of peri-implant bone tissue surrounding SLA and Ti (PLL/CA-3.0) implants, with and without exposure to *Porphyromonas gingivalis* and *Aggregatibacter actinomycetemcomitans*. Results demonstrate that the PLL/CA-3.0 coating (group F) enhances osteogenesis and antibacterial activity. (b) 3D micro-CT reconstruction of femurs with Ti implants coated with chitosan, collagen, and hydroxyapatite (CCH) shows significant osteolytic destruction in the Ti–OH + *S. aureus* group after 4 weeks, while only minor osteolysis occurred in the Ti-CCH + *S. aureus* group. (b′) Detection of bacterial colonization on implants and surrounding bone tissue using the spread plate method. (b″) Quantification of *S. aureus* adhered to implant rods and bone tissue at 4 weeks shows significantly fewer bacterial colonies in the Ti-CCH + *S. aureus* group compared to the Ti–OH + *S. aureus* group. (c) Evaluation of QCS/HA/LAP coating on new bone formation around implants. Granulation tissue was prevalent in the bare PEEK group, whereas the coated groups, particularly PEEK@QCS/HA/LAP, showed increased bone formation. Masson staining indicated that the LAP-intercalated coating promoted the maturation of new bone, with van Gieson and toluidine blue O staining confirming enhanced bone formation in coated implants at both 6 and 12 weeks, consistent with micro-CT findings (c′). (c″) *In vivo* implant-based soft tissue infection model showing reduced inflammation in QCS-containing groups (H&E staining) and decreased bacterial presence (Giemsa staining) in the PEEK@QCS/HA and PEEK@QCS/HA/LAP groups at days 3 and 7 postsurgery. Parts a and a′ reprinted and adapted with permission from John Wiley & Sons (License Number: 1526768–1). Parts b, b′, and b″ reprinted and adapted with permission from Elsevier (License Number: 5780370420377). Parts c, c′, and c″ reprinted under the terms of the Creative Commons CC BY license.

The development of LbL to drug delivery has garnered considerable attention within the dental implant field for the antimicrobial potential as a coating implant surface.^33^ However, many studies have focused on applying this technology to the implant itself, which poses challenges due to the mechanical forces and friction exerted during placement into the bone, potentially compromising the integrity of the coating, as discussed earlier.^152−155^ Contrarily to the prevention and treatment concepts, several articles have described the system as an interesting strategy to prevent infection (Figure 5). However, the multilayers built-up on a surface act as a structure to hold the drug, which means that the LbL needs to be responsive to different stimuli to enable the release of the drug.

The LbL system could be easily prepared on implant abutment surfaces. The fact that the system is considered fragile to mechanical action, which means that it could be easily removed during toothbrushing, does not invalidate its importance. The main idea of creating a stimulus-responsive system is to understand that it will be degraded. However, the lifetime depends on the anionic and cationic reagents used and could be manipulated to allow the system to remain on the surface for longer, according to the pharmacological requirement to treat the target disease.

Furthermore, implant abutments disclose a different set of challenges and opportunities for LbL coatings.^139^ Developing antimicrobial coatings for abutments requires specific considerations, such as to maintain the smoothness and hydrophilicity of the surface, ensure biocompatibility with soft tissues, prevent bacterial recolonization at the soft tissue interface, and promote proper tissue integration without compromising the peri-implant health.^139−141^ As such, the translation of LbL coatings for implant abutments remains a distinct and promising avenue of research, with the potential to significantly improve the clinical outcomes in peri-implantitis treatment. This is because the LbL coating does not affect the physical properties and can either maintain the original roughness or make the surface even more regular. With regard to the wettability, the reagents might also be selected to reduce the contact angle between surfaces and water and increase the hydrophilic requested property to abutment components.

Further advancements in this area would likely focus on optimizing the durability and efficacy of these coatings under the unique mechanical and biological conditions encountered by abutments and aligning preclinical testing models to reflect these specific needs. Several studies have shown the antimicrobial properties of LbL coatings *in vitro*, highlighting their ability to inhibit bacterial biofilm formation and reduce inflammatory responses around dental implants.^33,34^ However, translating these findings into human clinical practice requires robust preclinical testing in animal models and further characterization of the coatings.

Preclinical testing is a critical step in understanding how LbL coatings perform in biological environments. Most animal studies to date are targeting the application for implants rather than for abutments, and they have focused on small-animal models, such as rats and rabbits, which offer useful insights into biocompatibility and short-term antimicrobial efficacy.^152−155^ These studies have demonstrated significant reductions in bacterial colonization around the coated implants and provided preliminary evidence of biocompatibility with the surrounding hard tissues. To the best of our knowledge, with respect to abutment of dental implant applications, no comprehensive study has yet been conducted on the use of LbL antimicrobial coatings in animal models. While there is a growing body of research exploring LbL coatings for various biomedical applications,^33,127,156−161^ the translation of this technology is still stuck in the preclinical phase, mostly because there is a lack of focus on the conceptual issues involved in the creation process. Specifically for abutments, the preclinical animal studies remain limited. This gap underscores the need for further investigation into the efficacy, biocompatibility, and long-term performance of LbL coatings *in vivo*, especially in larger and more physiologically relevant animal models.

Collaboration between researchers and industry partners is essential for scaling up production and ensuring cost-effectiveness.^26^ Advances in manufacturing processes and materials are needed to make LbL coatings commercially viable. Ongoing research aims to refine LbL-coating technologies, optimize drug release mechanisms, and further validate their clinical efficacy.^121,130−134,147,148^ Innovations in coating materials and design, along with comprehensive clinical trials, will be critical for successful translation.^26^

## 7 Conclusion

A thorough comprehension of the working mechanism of Layer-by-Layer (LbL) assembly is crucial for researchers aiming to implement multilayer strategies within the dental implant field, particularly in the development of antimicrobial coatings to combat peri-implantitis. Current knowledge regarding the LbL system highlights its responsiveness to various chemical and physical stimuli. However, the responsive and degradable nature of the materials used in assembling the LbL system imposes limitations on its biomedical application in terms of the physical site and purpose. From a dental implant perspective, the fragility of the coating and its susceptibility to physical force necessitate the application of the LbL system onto abutment surfaces, which are in direct contact with soft tissue.

Moreover, the versatility of the LbL system in incorporating a wide range of drugs and its ability to respond to external stimuli make it a potential strategy for creating comprehensive antimicrobial coatings focused on treating peri-implantitis. The mechanism of action of the LbL system primarily involves the controlled release of drugs, justifying its use when the disease is already present. In contrast, for prevention purposes, where the aim is to deter the onset of the condition, a system with a short shelf life would not suffice.

Despite efforts to align clinical conditions with the nature of the LbL system to better indicate its use as a coating, the complexity of the oral environment—encompassing saliva, enzymes, varying pH levels, and brushing—remains a significant challenge to overcome.
